# Cumulative and co-occurring strains among mass public shooting perpetrators: implications for violence prevention

**DOI:** 10.1186/s40621-026-00690-5

**Published:** 2026-05-24

**Authors:** Jaclyn Schildkraut, Emily A. Greene-Colozzi, M. Hunter Martaindale

**Affiliations:** 1 Regional Gun Violence Research Consortium, Rockefeller Institute of Government, Albany, NY USA; 2 School of Criminology and Justice Studies, University of Massachusetts Lowell, Lowell, MA USA; 3 School of Criminal Justice and Criminology, Texas State University, San Marcos, TX USA

**Keywords:** Mass public shootings, Violence prevention, Targeted violence, Risk factors, Strain, Behavioral warning signs, Threat assessment, Cumulative risk

## Abstract

**Background:**

Mass public shootings represent a significant source of fatal and nonfatal injury in the United States. This study examines patterns of strain among individuals who carried out these events, with attention to the prevalence, co-occurrence, and cumulative burden of stressors.

**Methods:**

Data were drawn from a database of mass public shootings in the United States from 1999 to 2024, comprising 171 incidents and 175 perpetrators. Information was collected using a structured, multi-source approach including official records, secondary reports, and triangulated media sources. Eighteen dichotomous indicators of strain were coded. Descriptive statistics assessed prevalence and cumulative strain, and phi coefficients examined pairwise associations.

**Results:**

Perpetrators experienced multiple, overlapping forms of strain (mean = 4.78; SD = 2.78). Psychiatric concerns, job-related difficulties, and interpersonal stressors were most common. Strains frequently co-occurred, with strong associations among interpersonal and psychological factors and between structural stressors and ideological motivations. Most perpetrators experienced multiple concurrent stressors, indicating that these events rarely are preceded by a single, isolated grievance.

**Conclusions:**

Mass public shootings may be better understood as the result of cumulative and co-occurring strains rather than isolated risk factors. Injury and violence prevention efforts may be strengthened by emphasizing patterns of stressors, early identification, and coordinated, systems-level responses, including threat assessment.

## Background

Mass public shootings remain a persistent public health concern in the United States, resulting in significant loss of life, nonfatal injury, psychological trauma, and long-term disruption to affected communities [1, 2]. Beyond the immediate casualties, these events generate widespread fear and impose substantial social and economic costs [1, 3]. These effects often extend beyond directly impacted communities, influencing perceptions of safety, institutional trust, and psychological well-being across broader populations [1, 3–5]. As such, identifying opportunities for prevention has become a critical priority for public health systems [1].

A growing body of research has examined the characteristics and antecedents of individuals who carry out mass public shootings [6, 7]. Prior studies have identified a range of stressors and warning behaviors, including interpersonal conflicts, employment and financial difficulties, experiences of rejection or marginalization, and mental health concerns [6–9]. Importantly, this work suggests that perpetrators rarely experience a single, isolated stressor [8, 9]. Rather, multiple grievances and life stressors often precede acts of targeted violence [8, 10]. Relatedly, threat assessment and targeted violence research has emphasized that concerning behaviors and warning signs frequently emerge in clusters and may escalate over time rather than appearing independently [11–15]. Prior research also has highlighted variation across types of mass public shooters, with differences in motivations, planning, and behaviors [6, 9]. These studies further suggest, however, that common social and psychological pathways involving cumulative strain may underlie these events across offender types [6, 9].

Despite these advances, comparatively less empirical attention has been devoted to systematically examining how stressors co-occur or accumulate across perpetrators within quantitative analyses. Prior threat assessment and targeted violence research has emphasized the importance of evaluating warning signs and stressors collectively rather than in isolation [11–13, 16]. Existing studies, however, still have focused primarily on identifying and cataloging individual risk factors and warning behaviors [17, 18], with comparatively fewer analyses examining broader patterns of cumulative and overlapping strain across perpetrators [7, 8]. As a result, there is limited empirical understanding of whether certain stressors tend to cluster together and how the cumulative burden of strain varies across individuals [7, 8].

Conceptually, these stressors can be understood as forms of strain, broadly defined as adverse experiences or perceived grievances that contribute to psychological distress [19]. More broadly, General Strain Theory posits that exposure to strain increases the likelihood of maladaptive coping responses, particularly when stressors are perceived as severe, unjust, or difficult to escape [19]. Relatedly, prior theoretical and empirical work has emphasized the importance of cumulative strain in understanding mass violence [10, 20]. Building on this framework, Levin and Madfis proposed a sequential model in which chronic (e.g., persistent social marginalization or failure), uncontrolled (e.g., instability across multiple life domains), and acute (e.g., triggering crises or losses) strains accumulate over time, ultimately contributing to the planning and execution of mass attacks [10].

Consistent with these perspectives, pathway and threat assessment frameworks suggest that concerning behaviors, grievances, and stressors may escalate and reinforce one another over time rather than emerge independently [12, 16, 21]. These frameworks further emphasize the importance of evaluating patterns of behaviors and stressors collectively rather than relying on isolated warning signs or singular risk factors [22, 23]. Collectively, this body of research suggests that mass public shootings may be better understood as the result of interacting and cumulative stressors rather than discrete precipitating factors alone. Empirical research systematically examining the cumulative and co-occurring nature of strain among mass public shooters, however, remains comparatively limited [5, 6, 20].

The present study addresses this gap by examining patterns of strain among individuals who carried out mass public shootings. Specifically, we (1) describe the prevalence of a range of identified grievances, (2) assess the extent to which these grievances co-occur, and (3) examine the cumulative burden of strain across perpetrators. By examining patterns of overlapping and accumulating stressors, this study seeks to advance empirical understanding of how strains cluster and interact among perpetrators of mass public shootings. In doing so, the findings may help inform injury and violence prevention efforts that move beyond isolated risk factors and instead consider the broader constellation of experiences that may signal elevated risk.

## Methods

### Data source and sample

Data for this study were drawn from a database of mass public shootings developed by the research team using a structured, multi-source data collection process. The database includes incidents occurring in the United States between 1999 and 2024 and was designed to systematically capture information on perpetrator characteristics, behaviors, and pre-attack experiences.

Mass public shootings were defined as incidents of targeted violence occurring in public or populated locations, involving one or more shooters and multiple victims (fatalities and/or injuries), within a single 24-hour period. Victims and locations were selected either at random or for symbolic value, and incidents associated with gang activity or organized terrorist actions were excluded [24]. Cases meeting these criteria were restricted further to those in which there was confirmed evidence of pre-attack leakage or warning behaviors, as established in prior research [25]. This resulted in a final sample of 171 incidents involving 175 perpetrators, including four cases with multiple offenders.

### Data collection and coding procedures

Data were collected using a three-tiered approach designed to maximize completeness and reliability of available information. Tier 1 sources consisted of official records, including law enforcement documents and court records, obtained through systematic public records requests. In total, more than 280 requests were submitted, yielding over 143,000 pages of official documentation.

Tier 2 and Tier 3 sources were identified through structured open-source searches using targeted keywords across 29 search engines and media platforms. Tier 2 sources included secondary summary and interpretive materials, such as after-action reports, case studies, and agency press releases. Tier 3 sources consisted of media reports, which were triangulated across at least three independent, reputable outlets for each variable to ensure accuracy and completeness. The full database consisted of more than 153,500 pages of records across the three tiers.

Each case was initially coded by a trained research assistant using a standardized coding instrument and operational definitions. Cases then were independently reviewed by one of the study authors. Following this review, a coding verification memo documenting coding decisions and supporting source material was prepared and evaluated by a second author. Any discrepancies identified during the review process were discussed and reconciled through consensus, and coding decisions were updated prior to final inclusion in the dataset. In limited cases involving unresolved differences in interpretation, a third study author served as an adjudicator.

### Measures

The present study focuses on 18 dichotomous indicators of strain, representing a range of stressors, grievances, and motivations identified in the literature, including analyses of targeted violence [12–14]. Psychiatric concerns included documented mental health symptoms, diagnoses, psychiatric crises, suicidality, prior mental health treatment or contact, and other evidence of psychological distress documented in available records. Job-related difficulties included documented employment instability, disciplinary action, job loss, or workplace conflict, while familial troubles included family conflict, estrangement, loss, or other significant interpersonal disruptions within the family context. Each indicator was coded as present (1) when sufficient evidence was available, or absent (0) when confirmatory evidence indicated that the strain was not present. In cases where available information was insufficient to determine whether a strain was present, the variable was coded as missing.

A cumulative strain index was created by summing the number of strain indicators present for each perpetrator, resulting in a count variable representing the total number of identified strains. Higher values on this index indicate greater exposure to multiple, co-occurring stressors.

### Analytic approach

Descriptive statistics were used to examine the prevalence of individual strain indicators and the distribution of cumulative strain across perpetrators. Pairwise associations between strain indicators were assessed using phi coefficients, which are appropriate for dichotomous variables. Analyses were conducted using pairwise deletion to account for missing data.

Confirmatory evidence of absence was relatively uncommon for several variables, resulting in limited variability and preventing the estimation of some pairwise associations. Accordingly, analyses focused on interpretable associations based on available data.

## Results

The sample consisted of 175 perpetrators involved in 171 mass public shooting incidents. The majority of perpetrators were male (94.9%) and White (62.3%). Ages ranged from 12 to 88 years (mean = 32.9 years). Incidents most frequently occurred in schools (24.6%) and workplaces (21.6%).

### Prevalence of strain indicators

Table 1 presents the prevalence of identified strain indicators among perpetrators. Psychiatric concerns were the most frequently observed strain (64.6%), followed by job-related difficulties (51.4%), familial troubles (50.3%), and relationship problems (41.1%). School-related difficulties (34.3%), bullying (30.3%), and financial strain (26.9%) also were commonly identified. Less frequently observed strains included ideological motivations (18.3%), fame-seeking (16.6%), religious grievances (12.0%), and homophobia (8.6%).

### Co-occurring strain

Analyses of pairwise associations revealed several patterns of co-occurring strain (Table 2). Interpersonal stressors were strongly interrelated, with relationship problems and familial troubles exhibiting a perfect association in cases with complete data (φ = 1.00, *p* < .001), and both demonstrating moderate associations with financial strain (φ = 0.55–0.56).

Interpersonal strains also were closely linked to psychiatric concerns. Relationship and familial difficulties each showed strong positive associations with psychiatric concerns (φ = 0.70, *p* < .001), and financial strain similarly was associated with psychiatric concerns (φ = 0.56, *p* < .001).

Additional associations connected structural stressors to ideological motivations. Job-related difficulties were moderately associated with ideological motivation (φ = 0.55, *p* = .006), and financial strain also demonstrated a positive association with ideological motivation (φ = 0.67, *p* = .024). These estimates were based on smaller subsamples, and limited variability in some indicators prevented the estimation of several additional pairwise associations.

### Cumulative strain

**Fig. 1:**
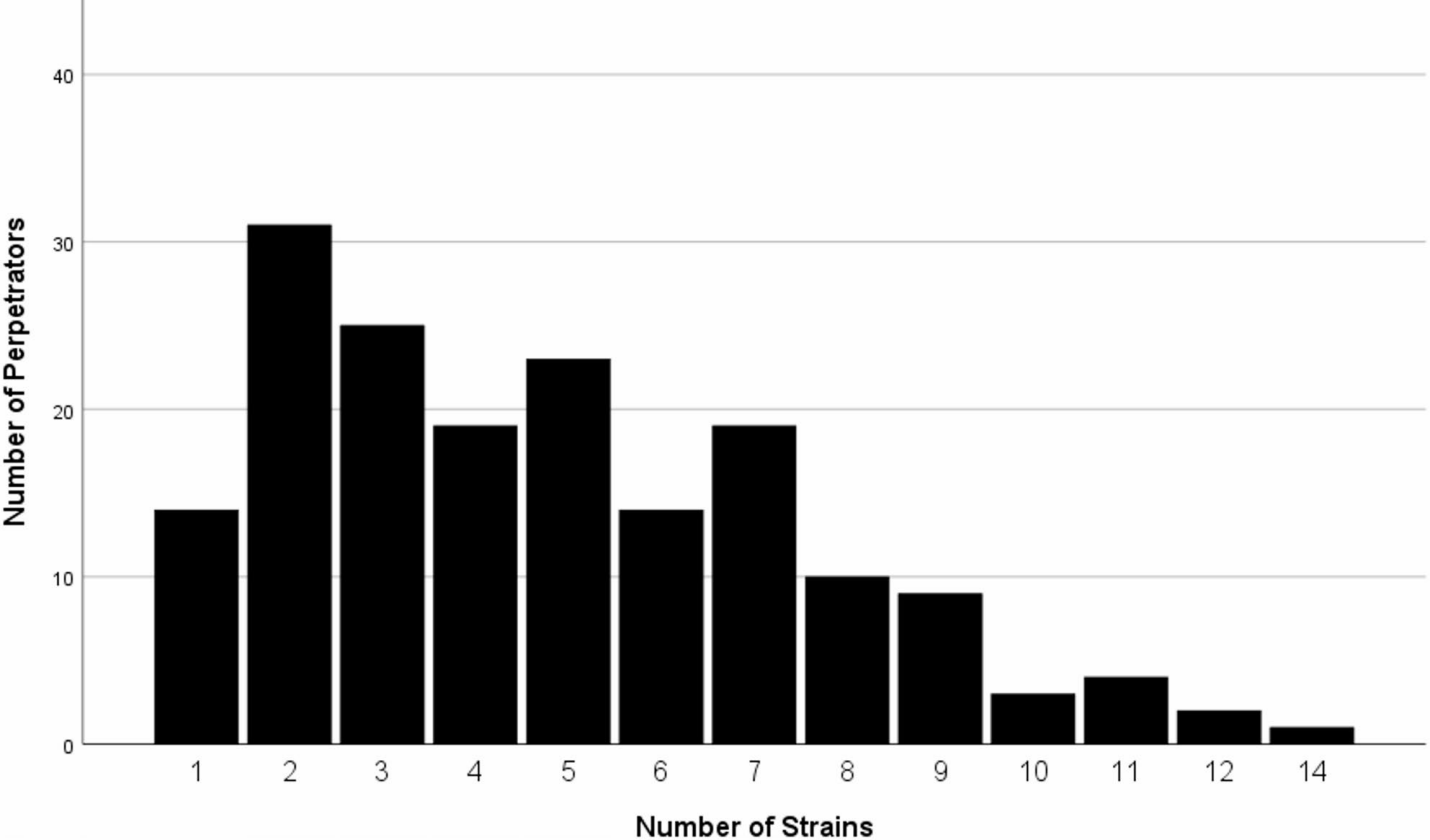
Distribution of Cumulative Strain Among Perpetrators (*N* = 175)

Perpetrators experienced multiple strains, with a mean cumulative strain score of 4.78 (SD = 2.78) and a median of 4. The number of identified strains ranged from 1 to 14, with the majority of perpetrators (74.9%) experiencing between 2 and 7 stressors. As shown in Fig. 1, the distribution of cumulative strain indicates that perpetrators typically experienced multiple, co-occurring grievances rather than isolated stressors.

## Discussion

This study examined patterns of strain among individuals who carried out mass public shootings, with particular attention to the co-occurrence and cumulative burden of stressors. The findings indicate that perpetrators experienced multiple, overlapping forms of strain rather than isolated grievances. Several types of strain were especially prevalent, including psychiatric concerns, employment-related difficulties, and interpersonal stressors such as family and relationship problems. These strains also frequently co-occurred, with particularly strong associations observed among interpersonal and psychological factors. These findings suggest that mass public shooters are characterized not by a single precipitating factor, but by a combination of overlapping stressors that accumulate over time.

A central finding of this study is that perpetrators experienced cumulative strain rather than isolated stressors, highlighting the limitations of single-factor explanations for mass public shootings. Perpetrators commonly experienced multiple forms of strain, many of which co-occurred. At the same time, the distribution of cumulative strain demonstrated substantial variability across perpetrators, underscoring the heterogeneity of pathways leading to mass public shootings. This pattern is consistent with prior theoretical work suggesting that different forms of strain, including chronic, uncontrolled, and acute stressors, accumulate and interact over time in ways that contribute to mass violence [10]. This finding also aligns with broader theories of strain, which posit that exposure to multiple stressors can increase pressure on individuals and heighten the likelihood of extreme behavioral responses [19].

At the same time, these findings should not be interpreted to suggest that cumulative strain alone is sufficient to explain acts of mass public violence. Access to firearms and other facilitating factors also likely play an important role in determining whether individuals are capable of carrying out acts of mass violence. Broader contextual influences, including differences in firearm policy and regulatory environments across states, also may shape how cumulative strain and behavioral risk factors manifest in acts of targeted violence. As such, strain should be understood as one component within a broader constellation of individual, situational, and structural factors associated with these events. These findings provide empirical support for cumulative strain frameworks and underscore the importance of understanding mass public shootings as the result of multiple, overlapping stressors rather than a single precipitating factor.

In addition to the cumulative burden of strain, the findings also highlight patterns of co-occurring stressors. As these results indicate, strains were not experienced independently among perpetrators, and not all stressors co-occurred consistently or randomly. The strongest cluster of co-occurring strains involved psychiatric and interpersonal factors, particularly family and relationship troubles or loss. Perpetrators who experienced these interpersonal stressors also commonly experienced psychiatric difficulties, which may have limited their ability to cope with these events. Another pattern emerged between structural and ideological strains, such that perpetrators experiencing job-related or financial difficulties also were more likely to exhibit ideological motivations that could reframe the sources of these experiences. These findings underscore the importance of examining not only the number of strains present but also how they co-occur, as these patterns may shape how strain manifests in mass violence.

Taken together, the findings of this study have important implications for how risk of violence and injury is understood, identified, and assessed. Current approaches often rely on a checklist mentality that focuses on individual warning signs and risk factors. The findings also are consistent with pathway and threat assessment frameworks, including work by the U.S. Secret Service National Threat Assessment Center (2019, 2021, 2023), which emphasize that acts of targeted violence often are preceded by patterns of cumulative and interacting stressors, concerning behaviors, and warning signs rather than singular indicators in isolation. As a result, these approaches may overlook patterns and clusters that reflect a cumulative burden of strain, increasing the likelihood that individuals respond to these pressures through extreme violence. Understanding this collective burden may improve the identification of individuals at risk for mass public shootings and support earlier intervention. It also may strengthen prevention efforts by informing systems-level responses, such as threat assessment approaches.

This study is subject to several limitations that warrant acknowledgment. First, data were derived from available records, which varied in completeness across cases. Although efforts were made to ensure cases were as complete as possible, it is possible that the prevalence of some stressors in the present study is underestimated. In addition, although the dataset was developed through a structured multi-stage verification and reconciliation process, formal interrater reliability statistics were not calculated as part of the original coding framework. Second, the lack of confirmatory evidence that stressors were not present, combined with missing data and differences in the availability of information across strain indicators, limited the ability to estimate associations between some strains. As a result, additional significant associations between strains may not have been captured. Third, because this study focused on mass public shootings, and specifically on cases in which perpetrators demonstrated observable warning behaviors and communications, the findings may not be generalizable to other forms of violence. Finally, the absence of a comparison group limits the ability to assess whether similar patterns of strain are present among individuals who do not engage in mass violence. Future research may benefit from examining how psychiatric concerns, behavioral histories, and cumulative strain interact with attack-related characteristics and outcomes across perpetrators. In addition, person-centered analytic approaches may help identify distinct constellations or profiles of strain among perpetrators and further clarify heterogeneity in pathways to mass public shootings.

## Conclusions

These findings have important implications for injury and violence prevention. Practitioners should move beyond relying solely on individual warning signs and instead assess patterns of stressors across domains. In practice, this means attending to combinations of interpersonal, psychological, and structural stressors and considering their cumulative burden. This approach may help identify individuals at risk earlier and support more timely and targeted intervention. Integrating these considerations into existing assessment processes can strengthen prevention efforts and inform coordinated, systems-level responses. Public awareness also is critical, as individuals in the community often are the first to observe concerning behaviors. Educating the public to recognize patterns of cumulative stressors, rather than isolated warning signs, may improve reporting and help bring individuals of concern to the attention of practitioners.

Effective prevention also requires coordinated, systems-level approaches. This includes the involvement of multiple stakeholder groups, such as education, workplaces, law enforcement, and mental health. Interdisciplinary coordination is essential for effective threat assessment and management, as is ensuring that team members have access to training and resources to recognize patterns of stressors that may precede mass violence. Policymakers can support these efforts by ensuring the availability of training and by investing in the development and sustainability of these teams’ infrastructure through funding and other resources. Together, these approaches underscore the importance of coordinated, multi-level strategies to prevent mass public shootings.

**Table 1:** Prevalence of Identified Strain Indicators Among Perpetrators (*N* = 175)

Strain Indicator	*n*	%
Psychiatric concerns	113	64.6
Job troubles / loss	90	51.4
Familial troubles / loss	88	50.3
Relationship troubles / loss	72	41.1
School troubles / loss	60	34.3
Bullying	53	30.3
Financial troubles / loss	47	26.9
Conflict with friends / peers	44	25.1
Trauma / abuse	39	22.3
Social rejection	37	21.1
Inadequacy	37	21.1
Racism	34	19.4
Ideological motivation	32	18.3
Fame-seeking	29	16.6
Religious grievance	21	12.0
Homophobia	15	8.6
Misogyny	13	7.4
Other	8	4.6

Percentages are based on the total sample (*N* = 175). Indicators were coded as present when sufficient evidence was available; absence was coded only when confirmatory evidence indicated the strain was not present. Cases lacking sufficient information were coded as missing

**Table 2:** Pairwise Associations Between Selected Strain Indicators Among Perpetrators

Strain Pair	φ	*p*-value	*n*
Relationship problems – Familial troubles	1.00	< 0.001	40
Relationship problems – Psychiatric concerns	0.70	< 0.001	52
Familial troubles – Psychiatric concerns	0.70	< 0.001	69
Financial strain – Psychiatric concerns	0.56	< 0.001	35
Relationship problems – Financial strain	0.56	0.002	29
Familial troubles – Financial strain	0.55	0.004	25
Job-related difficulties – Ideological motivation	0.55	0.006	23
Financial strain – Ideological motivation	0.67	0.024	11

φ = phi coefficient. Values reflect pairwise associations between dichotomous indicators. Analyses were conducted using pairwise deletion; therefore, sample sizes vary across comparisons. Only statistically significant and interpretable associations are shown

## Data Availability

The datasets used and/or analyzed during the current study are available from the corresponding author on reasonable request.
